# Comparison of detection methods to estimate asexual *Plasmodium falciparum* parasite prevalence and gametocyte carriage in a community survey in Tanzania

**DOI:** 10.1186/1475-2875-13-433

**Published:** 2014-11-18

**Authors:** Felista Mwingira, Blaise Genton, Abdu-Noor M Kabanywanyi, Ingrid Felger

**Affiliations:** Swiss Tropical and Public Health Institute, Socinstrasse 57, 4002 Basel, Switzerland; University of Basel, Petersplatz 1, 4002 Basel, Switzerland; Dar Es Salaam University College of Education, PO Box 2329, Dar Es Salaam, Tanzania; Ifakara Health Institute, PO Box 78373, Dar Es Salaam, Tanzania

**Keywords:** *Plasmodium falciparum*, Gametocyte, Prevalence, Quantitative PCR, *pfs25*, Light microscopy

## Abstract

**Background:**

The use of molecular techniques to detect malaria parasites has been advocated to improve the accuracy of parasite prevalence estimates, especially in moderate to low endemic settings. Molecular work is time-consuming and costly, thus the effective gains of this technique need to be carefully evaluated. Light microscopy (LM) and rapid diagnostic tests (RDT) are commonly used to detect malaria infection in resource constrained areas, but their limited sensitivity results in underestimation of the proportion of people infected with *Plasmodium falciparum*. This study aimed to evaluate the extent of missed infections via a community survey in Tanzania, using polymerase chain reaction (PCR) to detect *P. falciparum* parasites and gametocytes.

**Methods:**

Three hundred and thirty individuals of all ages from the Kilombero and Ulanga districts (Tanzania) were enrolled in a cross-sectional survey. Finger prick blood samples were collected for parasite detection by RDT, LM and molecular diagnosis using quantitative *18S rRNA* PCR and *msp2* nPCR. Gametocytes were detected by LM and by amplifying transcripts of the gametocyte-specific marker *pfs25*.

**Results:**

Results from all three diagnostic methods were available for a subset of 226 individuals. Prevalence of *P. falciparum* was 38% (86/226; 95% CI 31.9–44.4%) by qPCR, 15.9% (36/226; 95% CI 11.1–20.7%) by RDT and 5.8% (13/226; 95% CI 2.69- 8.81%) by LM. qPCR was positive for 72% (26/36) of the RDT-positive samples. Gametocyte prevalence was 10.6% (24/226) by *pfs25*-qRT-PCR and 1.2% by LM.

**Conclusions:**

LM showed the poorest performance, detecting only 15% of *P. falciparum* parasite carriers identified by PCR. Thus, LM is not a sufficiently accurate technique from which to inform policies and malaria control or elimination efforts. The diagnostic performance of RDT was superior to that of LM. However, it is also insufficient when precise prevalence data are needed for monitoring intervention success or for determining point prevalence rates in countrywide surveillance. Detection of gametocytes by PCR was 10-times more sensitive than by LM. These findings support the need for molecular techniques to accurately estimate the human infectious reservoir and hence the transmission potential in a population.

## Background

Records of Tanzanian malaria indicator surveys show a general decline in malaria prevalence among children under five years of age, from 18% in 2008 to 9% in 2012 [1, 2]. This decline has been attributed to countrywide implementation of malaria interventions, including indoor residual spraying (IRS), mass distribution of insecticide-treated nets (ITNs), long-lasting ITNs and the use of artemisinin-based combination therapy (ACT), which effectively kills both asexual blood stage parasites and immature gametocytes, thereby reducing transmission [3, 4].

Early diagnosis and prompt treatment are essential for appropriate malaria management. The World Health Organization (WHO) recommends laboratory confirmation of malaria before treatment, either by microscopy or by immuno-chromatographic rapid diagnostic test (RDT) [5]. Accurate malaria diagnosis is not only important for case management but also for estimating parasite prevalence in community surveys. Light microscopy (LM) is a standard tool for malaria diagnosis in resource constrained areas such as Tanzania. However, its performance is limited due to a lack of expertise and its low limit of detection (LOD) of about 50 parasites/μL of blood, which does not allow detection of low parasite densities [6, 7]. Although expert microscopists can attain a LOD of around 20 parasites/μL of blood [8], such high sensitivity is hardly ever achieved in field settings. RDTs are easier to use and their sensitivity is comparable to that of LM in the field [9, 10]. Currently, RDTs are widely used in community surveys but, owing to a low LOD, their performance in low endemic field settings is limited [11].

Recently, molecular tools for parasite detection have been introduced in many laboratories in endemic countries and are increasingly applied in monitoring interventions and epidemiological field surveys [12–14]. These molecular assays have LODs between 0.34–0.002 parasites/μL of blood, which results in more sensitive and reliable parasite detection. Due to their higher sensitivity, PCR-based techniques can be used to assess the extent to which parasite prevalence has been underestimated in endemic settings such as Tanzania, where malaria prevalence is routinely measured by classical LM [1, 15–17], complemented in recent years by RDTs [2]. So far, only a few studies in Tanzania have applied molecular techniques for blood stage parasite detection and even fewer for gametocyte detection [18–21]. Therefore, this study aimed to compare *P. falciparum* parasite and sexual stage prevalence rates as determined by LM and RDT with those obtained using molecular techniques, thereby assessing the usefulness of these different methods for epidemiological studies in Tanzania.

## Methods

### Study site and design

The study was conducted in the Kilombero and Ulanga (K-U) districts in Morogoro region in south-east Tanzania. The Ifakara Demographic Surveillance System (IHDSS) covers the study area [22]. The districts are primarily rural. Transmission of malaria is perennial with two rainy periods: from October to December and from March to May. The K-U districts were among the first areas in Tanzania to implement several malaria intervention strategies. The Kilombero Net project (KINET) successfully distributed ITNs, attaining 91% coverage by late 2000 [23]. This programme led to a four-fold reduction in entomological inoculation rates (EIR) [24] to about 78 infectious bites per year [25].

This study was conducted as an extension of the artemether-lumefantrine in vulnerable patients: exploring health impacts (ALIVE) project. Its main aim was to assess the impact of introducing ACT as a first-line anti-malarial treatment on all-cause mortality in infants/children under five years in the K-U districts.

A cross-sectional survey was performed between May and August 2011. Randomly selected households within the IHDSS were surveyed. A subset of 330 randomly selected individuals of all ages was included in the molecular analysis. The study was granted ethical clearance by the Ifakara Health Institute (IHI) reference number: IHI/IRB/AMM/10-2011 and by the National Institute for Medical Research Tanzania reference number: NIMR/HQ/R8c/Vol. I/184.

### Blood collection and sample storage

Finger prick blood was used to diagnose malaria positivity by (i) RDT SD Bioline Pan-pLDH/Pf-HRP2, (ii) blood smear and LM and (iii) PCR-based molecular diagnosis. Approximately 50 μL of whole blood were collected on Whatman® grade-3 filter paper, air dried in the field and stored at ambient temperature in separate sealed plastic bags with desiccant. Two blood spots on filter paper were prepared per individual, one of which was put in 300 μL TRIzol® (Invitrogen) to stabilize RNA and stored at -80°C. Samples in TRIzol® were shipped by air on refrigerant gel packs to the laboratory responsible for DNA and RNA extraction. RNA was extracted from 330 samples using the Qiagen RNeasy Plus ® protocol with on-column DNase digestion, to ensure removal of genomic DNA (gDNA) as described elsewhere [14]. RNA was stored at -20°C for a maximum of two weeks prior to cDNA synthesis and amplification. One additional blood spot per patient was air-dried and preserved in a sealed plastic bag with desiccant at -20°C until shipped at room temperature. DNA was extracted from 226 dried blood spots using the Chelex protocol [26]. DNA was stored at -20°C for one to two weeks until used in PCR.

### Microscopy blood smear reading

Thick and thin blood films were prepared in the field, air dried, Giemsa-stained and read for detection and quantification of malaria parasites according to Standard Operating Procedures at the IHI laboratory. Asexual parasites were reported out of 200 leukocytes. Gametocyte detection by LM was based on a volume of blood corresponding to 500 leucocytes. Assuming 8,000 leucocytes/μL blood, parasite density (expressed as parasites per μL blood) was calculated by multiplying LM counts by a factor of 40 if parasites were reported out of 200 leukocytes or by 16 for 500 leukocytes. Two independent qualified technicians read all slides. In case of discrepancy between two readers, a third reader was requested. The final result was the mean of the two closest readings out of three. For cases of positive/negative discrepancy the majority decision was adopted.

### Molecular assays

A qPCR targeting the *P. falciparum* S-type 18S rRNA genes was performed on all DNA samples to determine parasite prevalence [27]. As a reference, a nested PCR (nPCR) targeting the merozoite surface protein 2 (*msp2*) was performed on all DNA samples [28]. Gametocytes were detected by amplifying transcripts of the gametocyte-specific expressed marker *pfs25* [14]. *pfs25* transcripts were reverse transcribed and the resulting *pfs25* cDNA was amplified by qPCR. The RNA-based quantitative reverse transcriptase PCR (qRT-PCR) assay was performed on all extracted RNA samples after complete gDNA removal had been confirmed by a qPCR assay targeting 18S rRNA genes of all *Plasmodium species* [14]. To quantify *P. falciparum* parasites and gametocytes, copy numbers of the respective template per μL blood were calculated using standard curves obtained from assay-specific plasmids routinely included on each 96-well qPCR plate.

### Data analysis

All data was entered and analysed by STATA® version 13, Texas, USA. To compare the performance of different diagnostic tests, concordance of results was recorded. Parasite density/μL blood and marker-specific template copy number/μL blood were converted to log_10_.

## Results

This community survey included 330 individuals, the mean age was 18 years with an age range of 1–81 years. Of these, 21% were children <5 years, 44% were between 5–19 years. Individuals between 20–59 years and adults >60 years accounted for 30% and 4.5% of recruited individuals, respectively. A complete dataset including all four diagnostic methods was obtained for 226 participants and used to compare test performance.

### *Plasmodium falciparum*prevalence and density

**Figure 1:**
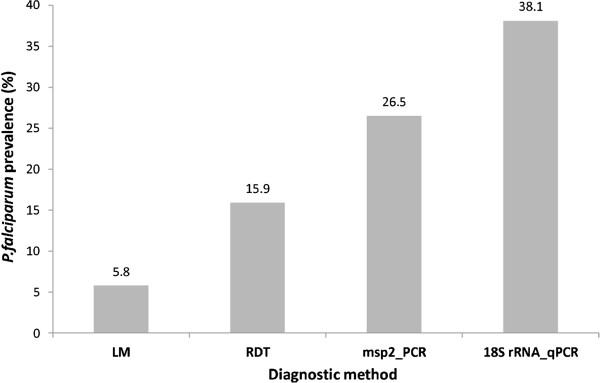
***Plasmodium falciparum*** **prevalence rates by LM, RDT,** ***msp2*** **nested PCR and** ***18S rRNA*** **qPCR performed in N = 226 samples from the Kilombero-Ulanga districts in Tanzania.**

Prevalence of *P. falciparum* blood stages in the K-U districts was 38% (86/226; 95% CI 31.9–44.4%) by *Pf18S rRNA* qPCR. A lower parasite prevalence of 26.6% (60/226; 95% CI 19–31.2%) was observed when *msp2* nPCR was performed. Of *msp2* positive samples, 83.3% (50/60) were confirmed by *Pf18S rRNA* qPCR. Only 58% (50/86) of *Pf18S rRNA* qPCR-positive samples were positive by *msp2* nPCR (Table 1). Thus, sensitivity of qPCR was superior to that of standard nPCR (Figure 1).

**Table 1:** **Comparison of the two molecular methods** ***Pf18S rRNA*** **qPCR and** ***msp2*** **nested PCR for** ***P. falciparum*** **parasite detection**

	***msp2***			
**18S rRNA qPCR**		Positive	Negative	Total
	Positive	50	36	86
	Negative	10	130	140
	Total	60	166	226

Pearson chi^2^ (1) = 71.0492 Pr = 0.000.

*Plasmodium falciparum* prevalence was 15.9% [(36/226; 95% CI 11.1–20.7%) by RDT and 5.8% (13/226; 95% CI 2.69- 8.81%) by LM. RDT was positive for 8/13 (61.5%) and qPCR for 11/13 (84.6%) LM-positive samples. Only 2/13 (15.4%) LM-positive samples were unidentified by both RDT and qPCR, suggesting that these two LM results were false positives (Table 2). Of 36 RDT-positive samples, 26 (72.2%) were also positive by qPCR, whereas the remaining 27.7% of RDT-positive samples were negative by qPCR and LM.

**Table 2:** **Concordance among three different diagnostic methods for detecting** ***P. falciparum*** **positivity**

Patterns of test positivity by three diagnostic methods			
18S rRNA qPCR (N _pos_= 86)	RDT (N _pos_= 36)	LM (N _pos_= 13)	total positive samples N = 98
+	-	-	57
+	+	-	18
-	+	-	10
+	+	+	8
+	-	+	3
-	-	+	2

**Figure 2:**
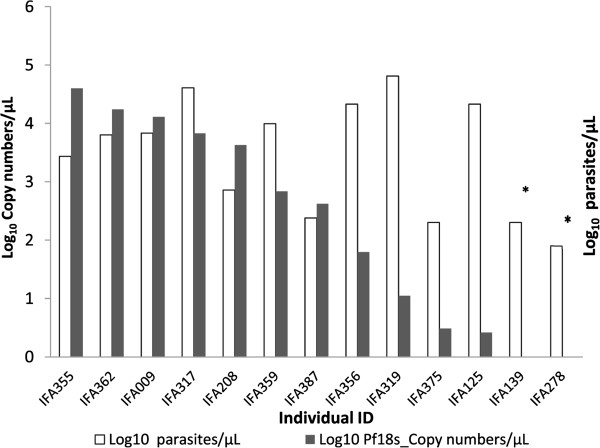
**Comparison of log**_**10**_ ***Plasmodium falciparum 18S rRNA*** **gene copy numbers/μL blood by qPCR and log**_**10**_ **parasite counts/μL blood by LM.** *Two LM-positive samples were negative by RDT and molecular assays and likely represent false positive microscopy results.

LM recorded a mean of 13,483 parasites/μL blood (range 80 to 64,640). *Pf18S rRNA* qPCR detected a mean of 6,524 *18S rRNA* gene copies/μL blood (range 0.9 to 155,293). *18S rRNA* copy numbers were not converted into parasite counts because trend-line experiments using ring stage parasites were not performed for filter paper blood spots with similar storage conditions. Moreover, original blood spots slightly varied in size and thus whole blood content also varied. A non-linear correlation was observed between log_10_ parasite density by LM and log_10_*18S rRNA* gene copy numbers/μL blood for all microscopy positive samples (Figure 2).

### Gametocyte prevalence

Gametocyte prevalence was determined by LM and qRT-PCR in 226 samples. Gametocyte carriage in the study population was 10.6% (24/226; 95% CI 6.6–14.7%) by qRT-PCR and 1.2% (3/226; 95% CI0.2–2.8%) by LM. Two of the three gametocyte carriers identified by LM were confirmed by molecular gametocyte detection. A large proportion of gametocytaemia (87.5%; 21/24) was submicroscopic.

The proportion of molecularly-identified gametocyte carriers among *P. falciparum* positive individuals is listed in Table 3 for all four independent diagnostic tests (LM, RDT, *msp2* nPCR and *18S rRNA* qPCR). In total, 3/13(23%) LM-positive and 9/36 (25%) RDT-positive *P. falciparum* infections harboured gametocytes detected by *pfs25* qRT-PCR. Among individuals deemed positive by *msp2* 12/60 (20%) carried gametocytes. This proportion was slightly higher than in individuals deemed positive by the more sensitive *18S rRNA* qPCR, with only 16.2% (14/86) of infections harbouring gametocytes.

**Table 3:** **Proportion of** ***P. falciparum*** **gametocyte carriers among individuals deemed positive by RDT, LM, or molecular assays (** ***18S rRNA*** **qPCR and** ***msp2*** **nested PCR)**

Malaria diagnosis	Gametocyte positive among ***Pf.***positive samples (% Gametocyte carriage among ***Pf***. positives)	Gametocyte positive by molecular ***Pfs25-***qRT-PCR	Non gametocyte carriers
LM	3/13 (23%)	3	10
RDT	9/36 (25%)	9	27
*msp2* nPCR	12/60 (20%)	12	48
*18S rRNA* qPCR	14/86 (16%)	14	72

## Discussion

Accurate estimation of malaria burden after implementation of effective malaria control programmes is of particular importance for evaluating and planning further intervention strategies. Accuracy of the diagnostic tests applied and knowledge of their limitations are essential. Therefore, we evaluated the performance of LM and RDT, the routinely used methods for estimating *P. falciparum* prevalence in the community, and compared it with that of qPCR for determining parasite positivity. Our results highlight the poor sensitivity of LM and the high prevalence of submicroscopic infections. Malaria prevalence in the K-U districts is vastly underestimated, if detection is based on LM only.

In many parts of Tanzania, LM is still widely used as the standard parasite confirmation method because supply of RDTs is unreliable owing to stock-outs. With the increasing success of interventions and as a consequence of reduced clinical malaria, it becomes increasingly important to determine prevalence rates in the community to estimate the remaining malaria burden and to monitor the effect of sustained control measures. In this context, the sensitivity of the diagnostic method, which greatly influences prevalence determination, becomes increasingly important.

We observed a sevenfold difference between parasite prevalence estimated by qPCR and that estimated by LM. Other studies in Thailand, Myanmar [29, 30] and Malawi [31], as well as a systematic review [32] have also reported more than two- to fivefold difference in asexual stage parasite prevalence estimates between classical LM and molecular detection. Several limitations of LM have been documented [33], such as its dependency on the expertise of the reader, the method of slide preparation, staining and reading, and last but not least, its LOD of about 50 parasites/μL blood. The LOD ranges from 20–100 parasites/μL blood between expert and field microscopists. Thus, the high prevalence of submicroscopic infections in the K-U districts is not surprising, and even slightly higher than in studies done elsewhere. Such an abundance of submicroscopic infections is expected in areas where malaria transmission has recently been reduced successfully because parasite densities are controlled by acquired immunity of previously exposed individuals [34].

Our study revealed that two of the 13 LM-positive samples were negative by RDT and by both molecular assays. These LM-positive samples were likely false positives that may have resulted from erroneous thick smear reads, as has been documented in other studies [35]. Massive over-diagnosis of more than twenty-fivefold difference in the prevalence rates (i.e. 53% versus 2% prevalence) has been reported in a comparative study of routine and expert LM in Tanzania [6].

The molecular methods (nPCR and qPCR) applied in this study were slightly discordant in parasite detection. This difference can be explained by a lower sensitivity of *msp2* nPCR compared to the *18S rRNA* qPCR, which is likely due to its greater amplicon size and thus less efficient amplification. Moreover, compromised integrity of parasite DNA could also lead to more efficient amplification of the shorter *18S rRNA* amplicon. However, some samples which were positive using the marker *msp2* were negative by qPCR. In samples with very low parasite densities, a chance effect in the template distribution to one reaction but not to the other could account for such discrepant results. Alternatively, PCR inhibitors could potentially be present in a sample, which may affect the qPCR assay more than the nested PCR assay.

Our PCR data help to understand the difference between LM and RDT results in this study. The discrepancies are likely due to the low sensitivity of LM as well as to the residual HRP antigen that remains after a cleared infection [36, 37]. The LOD of RDTs is roughly comparable to LM in the field, although the last generation of RDTs showed a higher sensitivity than previous ones [38]. Moreover, RDTs performed better than LM in community surveys [12, 39]. To estimate the proportion of parasite infections undetected by LM and RDT, qPCR with a substantially higher sensitivity of up to 0.34 parasites/μL blood was applied. The use of qPCR in our study increased malaria prevalence twofold, which is similar to differences between PCR- and RDT-detected prevalence reported elsewhere [12, 35, 39, 40]. About 30% of RDT positive samples were negative by our most sensitive qPCR assay. Variability in the interpretation of RDT results may have contributed to the discordance between RDT and PCR. A direct comparison between the results of qPCR and RDT is generally problematic because these two tests do not detect the same target molecule: while qPCR detects DNA from circulating parasites, RDT detects circulating antigens; hence a 100% concordant result is not expected.

Another explanation for the discrepancies between RDT, LM and PCR results could be that RDTs are actually capturing gametocytes in the absence of asexual forms. pLDH is produced by live parasites including gametocytes [41]. In confirmed samples containing only *P. falciparum* gametocytes, RDT was positive in 72% of samples with high gametocyte density (>500 gametocytes/μL blood) compared to only 20.5% RDT positives in samples with low gametocytaemia(>200 gametocytes/μL blood) , suggesting that the presence of gametocytes can compromise RDT results [42]. Similarly, among the three samples in our study that were RDT positive but qPCR negative, all harbored gametocytes by *pfs25* qRT-PCR. This could indicate the presence of gametocytes in the absence of asexual forms. Negativity by qPCR in a gametocyte-positive sample could be explained by the presence of only three *18S rRNA* gene copies per parasite genome, whereas the numbers of *pfs25* transcripts are much higher [14]. Other molecular markers, specific for asexual parasite stages, would be needed to prove the absence of any asexual parasite.

In our study, the prevalence of gametocytes by *pfs25* qRT-PCR was ten times higher than that by LM, indicating a high proportion of submicroscopic gametocytaemia in the community. Based on the low gametocyte prevalence by LM in previous years and in the same population [43], much higher gametocyte prevalence had been anticipated, but was not confirmed until now. An even greater difference in gametocyte detection between LM and molecular analysis by Quantitative Nucleic Acid Sequenced-based Amplification (QT-NASBA) has been observed in a community survey in Tanzania (0.4% and 15% positivity, respectively) [44]. In malaria epidemiology, submicroscopic gametocytaemia is important. It has been shown that submicroscopic gametocyte carriage substantially contributes to the human infective reservoir for onward transmission to mosquitoes. These studies have shown that even microscopy negative individuals can infect mosquitoes [45, 46]. Therefore, the observed 10.6% gametocyte prevalence in our study population is likely to sustain malaria transmission in the presence of an efficient vector.

Over the seven-year course of malaria community surveys in the K-U districts, molecular data were generated only during the 2011 survey. Therefore, the longitudinal effect of interventions in the study area on *P. falciparum* prevalence rates can only be analyzed by classical diagnostic means. Previous LM data from the IHDSS recorded declining malaria prevalence within the K-U districts, from 25% in 2004 to 4.6% in 2009 [22, 45]. The qPCR-based prevalence rates obtained from the 2011 survey now provide a more precise picture of the malaria prevalence in the K-U districts and put the very low prevalence rate by LM into a new perspective. LM seems inadequate as a diagnostic tool for surveillance of parasite infections in Tanzania at a point when transmission intensity is shifting from high to low. The question remains whether RDT diagnosis should be considered a suitable alternative. This test has the advantage of allowing on-site treatment for symptomatic or asymptomatic individuals with positive RDT results. It also allows comparison of data from different areas and countries that still use conventional techniques. The future might be to use both, RDT for all individuals and PCR in a subsample, to better gauge the magnitude of underestimation of the parasite prevalence. Molecular techniques should be used especially in areas of very low endemicity, where elimination is the prime objective.

## Conclusions

Light microscopy showed the poorest performance for detecting both *P. falciparum* asexual parasites and gametocytes. This implies the presence of a large proportion of submicroscopic parasitaemia and gametocytaemia in the K-U districts, a phenomenon that is common in areas of recently declining transmission. RDT performed better than LM, as it detected almost half of the *P. falciparum* carriers identified by molecular tools. However, in light of the PCR results, the gain in sensitivity of RDT over LM was still modest. However, the use of RDT adds to our understanding of the real transmission level, in the sense that it can also detect recently cleared infections (treated or not) that are no more detectable by LM or PCR. Thus, using both tools, PCR and RDT, which together are able to detect actual parasitaemia plus recent infections, may provide the most precise information by which to assess the impact of interventions and to decide on the best control strategies. To reliably estimate the malaria reservoir in areas of high submicroscopic parasitaemia, molecular tools are clearly justified.
